# Discrimination of Gain Increments in Speech-Shaped Noises

**DOI:** 10.1177/2331216518820220

**Published:** 2019-01-18

**Authors:** Benjamin Caswell-Midwinter, William M. Whitmer

**Affiliations:** 1 Hearing Sciences—Scottish Section, Division of Clinical Neuroscience, University of Nottingham, Glasgow, UK; 2 School of Medicine, Dentistry, and Nursing, College of Medical, Veterinary, and Life Sciences, University of Glasgow, Glasgow, UK

**Keywords:** just-noticeable differences, hearing aids, hearing-aid gain, hearing-aid fitting

## Abstract

Frequency-dependent gain adjustments are routine in hearing-aid fittings, whether in matching to real-ear targets or fine-tuning to patient feedback. Patient feedback may be unreliable and fittings inefficient if adjustments are not discriminable. To examine what gain adjustments are discriminable, we measured the just-noticeable differences (JNDs) for level increments in speech-shaped noises processed with prescription gains. JNDs were measured in the better ears of 38 participants with hearing impairment using a fixed-level, same-different task. JNDs were measured for increments at six individual frequency-bands: a 0.25-kHz low-pass band; octave-wide bands at 0.5, 1, 2, and 4 kHz; and a 6-kHz high-pass band. JNDs for broadband increments were also measured. JNDs were estimated at *d’* of 1 for a minimally discriminable increment in optimal laboratory conditions. The JND for frequency-band increments was 2.8 dB excluding the 0.25-kHz low-pass band, for which the JND was 4.5 dB. The JND for broadband increments was 1.5 dB. Participants’ median frequency-band and broadband JNDs were positively correlated. JNDs were mostly independent of age, pure-tone thresholds, and cognitive score. In consideration of self-fitting adjustments in noisier conditions, JNDs were additionally estimated at a more sensitive *d’* of 2. These JNDs were 6 dB for bands below 1 kHz, and 5 dB for bands at and above 1 kHz. Overall, the results suggest noticeable fine-tuning adjustments of 3 dB and self-fitting adjustments of 5 dB.

## Introduction

Frequency-dependent gain is the hearing-aid parameter fundamental to restoring audibility to listeners with hearing loss. This parameter, which we will refer to as the frequency-gain response (FGR), is most commonly set by the application of a prescription formula to pure-tone thresholds. The FGR is then verified by real-ear measurements (REMs) which assess the gains delivered to the tympanic membrane, allowing the clinician to account for ear canal resonance properties and fitting software errors. While presenting speech-shaped noises (SSNs) or recorded speech, gains at frequency bands are adjusted to prescribed targets via hearing-aid fitting software. FGR curves based on fitting software commonly deviate from real-ear gains, and REMs help ensure that prescription targets are met (Aazh & Moore, 2007; Aazh et al., 2012; Leavitt et al., 2017).

This procedure does not guarantee objective benefit nor patient satisfaction. Pure-tone thresholds do not provide a comprehensive measure of hearing status; FGR curves which maximize objective benefit may depend on abilities such as suprathreshold loudness perception, frequency resolution, and even cognitive capacity (Amlani & Schafer, 2009). Prescription formulas are constructed on average data; while they can provide a competent starting fit, patients often have their own preferences and clinicians are required to make adjustments accordingly (Dreschler, Keidser, Convery, & Dillon, 2008; Jenstad, Van Tasell, & Ewert, 2003; Keidser, Dillon, Carter, & O’Brien, 2012; Kuk, 1999; Nelson, 2001).

Fine-tuning is the patient-centered practice of adjusting parameters following the initial-fit or REM, and is largely focused on the FGR (Anderson, Arehart, & Souza, 2018; Jenstad et al., 2003; Thielemans, Pans, Chenault, & Anteunis, 2017). Naturally, the literature largely examines controlled and systematic fine-tuning (such as adaptive fine-tuning or fine-tuning with ecological stimuli); this is different from the unstructured fine-tuning routinely performed in a quiet room to live voice. Troubleshooting adjustments are also commonly made after a period of real-world use in response to reports of hearing difficulty or poor sound quality. These practices are often protracted, and there is a lack of both scientific evidence and clinical guidelines on how to optimally adjust even fundamental parameters (Anderson et al., 2018). Moore, Alcantara, and Glasberg (1998) reported that an adaptive fine-tuning gain procedure led to greater subjective and objective benefit versus a manufacturer’s initial fit. Several studies report that it is valuable to gradually adjust the FGR to aid acclimatization and avoid overamplification with first-time hearing-aid users (Marriage, Moore, & Alcántara, 2004; Smeds, 2004). A pilot study by Cunningham, Williams, and Goldsmith (2001) reported no difference in objective and self-reported benefit between groups of first-time hearing-aid users who did and did not receive fine-tuning, suggesting that it is an inefficient practice. Saunders, Lewis, and Forsline (2009) similarly reported that fine-tuning had no effect on self-reported benefit or satisfaction, although objective benefit was not measured; it is possible that fine-tuning led to some objective improvement that was not noticeable by participants. Saunders et al. did however report some benefit in that participants who received fine-tuning wore their devices more. Self-adjustment technology is becoming increasingly available, allowing patients to instantly fine-tune their devices in highly acoustically variable environments (Keidser & Convery, 2016). Evidence suggests that these adjustments vary widely among listeners, although the effect of such adjustments on speech intelligibility is unclear (Boymans & Dreschler, 2012; Nelson, Perry, Gregan, & Van Tasell, 2018). The efficacy and implementation of fine-tuning and self-adjustment technology requires greater research. Even so, adjusting the FGR—whether matching to a target with REMs, fine-tuning the initial fit, or troubleshooting to follow-up complaints—is a key component of the fitting process.

There are no guidelines on the scale of adjustments that should be made when fitting the FGR. Clinical practice varies widely and hearing-aid fitting software permits gain adjustments at levels of 1 dB (or less) at a range of manipulable frequency handles. Fine-tuning could be unreliable and inefficient if patients are unable to discriminate FGR adjustments (frequency-specific or broadband changes in output). It is unlikely that an FGR adjustment of a magnitude less than what is discriminable will elicit authentic patient feedback. This problem could be particularly apposite for paired comparison fittings and self-fitting technologies, in which empirically different parameter alternatives could be perceptually indiscriminable, particularly when in acoustically noisy environments. If adjustments improve audibility, but are not noticeable, then self-reported outcome measures will unlikely reflect an improvement. Further, a patient may expect adjustments to be immediately noticeable, which may lead to dissatisfaction and nonuse if they are not (Demorest, 1984).

Even with REM adjustments, discrepancies occur between the real-ear and target gain (Munro, Puri, Bird, & Smith, 2016); current clinical guidelines suggest fitting gain to prescription targets within tolerances of ± 5 dB for octave bands ranging from 0.25 to 6 kHz (British Society of Audiology, 2018), although there is an absence of direct perceptual evidence on which to base these values. Therefore, it is important to investigate the just-noticeable differences (JNDs) for FGR adjustments.

Discrimination of spectral peaks in complex stimuli is most germane to discrimination of FGR adjustments. Turner and Holte (1987) compared the abilities of five participants with normal hearing and eight participants with hearing impairment to discriminate a second-formant peak (1.82 kHz, 0.65 kHz bandwidth) in the vowel /ɛ/ at a range of presentation levels. A JND of 4 dB was asymptotic at presentation levels of 40 dB SPL and above for participants with normal hearing. Four participants with hearing impairment performed similarly, and for several, discrimination improved with level. JNDs for the remaining participants with hearing impairment were greater than 9 dB, only improving to 5 dB with high-pass (HP) gain. Using three trained participants with normal hearing, Moore, Oldfield, and Dooley (1989) tested 1 - and 8-kHz peak discrimination in broadband noises. Stimuli surrounding the peak were either presented at 30 dB SPL (where level cues were present) or a random level between 24 and 36 dB SPL; 1-kHz and 8-kHz JNDs were 2.1 dB and 2.5 dB, respectively, for peaks with bandwidths 0.5 times their center frequency. Varying the presentation level of stimuli had no effect on the JNDs. The disparity in results between the two studies may be because of stimulus differences or differences in the psychophysical procedures; Moore et al. used a two-alternative forced-choice task (2AFC) whereas Turner and Holte used a 4AFC task.

Profile analysis is an experimental paradigm which tests the ability to discriminate variations in spectral shape, typically an increment of a single pure-tone component relative to background components (Green, 1988). Roving level across stimuli (within trials) is a fundamental technique employed to ensure that discrimination is performed on the basis of a change in spectral shape, rather than a change in the output of the auditory filter centered at the increment. The application of profile-analysis thresholds to FGR discrimination is therefore limited, as a gain increment at a frequency-band may be discriminated based on the output of the corresponding auditory filter or a change in spectral shape. Lentz and colleagues (2003, 2004) were one of the few to examine the effect of hearing impairment on profile analysis. A negative effect was only identified when presenting narrowly spaced stimuli. This suggests that the excitation patterns of broadened auditory filters are smoothed with this type of stimuli. Therefore, adjustments within spectrally dense stimuli such as speech-shaped noises or speech may be more challenging for listeners with hearing impairment to discriminate, although profile analysis has been seldom examined with complex stimuli. Furthermore, weighting strategies varied among participants, reflecting coding difficulties not quantified by the audiogram. These results suggest that the exact strategies used by listeners with hearing impairment to discriminate spectral shape likely vary from listeners with normal hearing.

There has been previous research into the broadband FGR adjustments required to elicit a differing percept. Dirks, Alstrom, and Noffsinger (1993) had nine participants with hearing impairment make same-different judgments on varying FGR curves. Judgments were based on speech intelligibility and sound quality attributes. Presented over earphones, FGR curves were adjusted from an National Acoustics Laboratory - Revised (NAL-R) prescription reference for speech in noise at 62 dB SPL with a 3-dB signal-to-noise ratio (SNR). Adjustments of at least 4 dB were required to elicit a different judgment for over half of participants. The remaining participants—generally with more severe impairments—could not differ between FGR curves with adjustments as high as 11 dB. In a similar pilot study, Jenstad et al. (2007) had 23 participants with hearing impairment complete loudness ratings and speech recognition tasks with devices adjusted in overall gain. Speech was presented at a baseline level of 60 dB SPL. A nominal adjustment of 4.5 dB (from the fitting software) was reported to be judged different. In a brief discussion, Byrne and Dillon (1986) examined data from a separate study (Byrne, 1986), which had 11 participants with hearing impairment compare FGR curves presented over headphones. An adjustment of 3 dB or more resulted in a statistically significant difference in intelligibility and pleasantness ratings, however, the exact methodology and analysis were not discussed in detail. Importantly, these studies reported values based on preference and intelligibility judgments, rather than discrimination tasks. Subjective attributes—such as pleasantness—are defined by a listener’s own experience, whereas discrimination, the perception of a physical change in a stimulus, is performance-based, and yields objective psychophysical measures. Previous study has shown that the minimum SNR adjustments required to elicit differing preferences and behavior changes are much greater than the minimum SNR adjustments that can be discriminated (McShefferty, Whitmer, & Akeroyd, 2016).

Previous psychophysical evidence on the ability to discriminate narrowband (⅓-½ octave) adjustments in a broadband stimulus varies from 2 dB for trained listeners with normal hearing (Moore et al., 1989) to 4 to 9 dB for listeners with hearing impairment (Turner & Holte, 1987). Previous clinical evidence from pilot and supplemental studies has inferred from preference and intelligibility judgments that the broadband JND for speech stimuli is 3 to 4 dB (Byrne & Dillon, 1986; Dirks et al., 1993; Jenstad et al., 2007). Because of the variation in method and result, it is not clear from the previous literature (a) what is the JND for frequency-band adjustments from prescribed gain, (b) if the JND is dependent on hearing loss, and (c) if the frequency-band JND is center-frequency dependent.

In the current study, we measured the JNDs for frequency-band increments to verify REM tolerances and ascertain discriminable step-sizes for adjusting the FGR. We also measured the JNDs for broadband increments, analogous to traditional level discrimination. Participants were either hearing-aid candidates, or users. We presented SSNs, a common test signal for REMs (British Society of Audiology, 2018; British Society of Audiology & British Academy of Audiology, 2007), and employed a same–different task, eliciting a judgment appropriate to the clinic. SSNs were presented over headphones in quiet to better ears.

We additionally examined age and cognitive ability as mediators of discrimination (Gatehouse, Naylor, & Elberling, 2006; Lunner, 2003). Working memory, the limited, temporary store of information, is a crucial cognitive ability for auditory processing, and is particularly compromised with age (Babcock & Salthouse, 1990). While examined with a variety of methods, there is evidence that working memory capacity can inform optimal parameter fitting (Akeroyd, 2008; Rönnberg, Rudner, & Lunner, 2011). Lunner (2003) reported that hearing-aid users with high working-memory capacity were better at identifying the effects of amplification schemes varying in gain and compression than hearing-aid users with low working-memory capacity. There is also evidence that auditory discrimination is mediated by working memory, although these studies tested adults much younger than a typical audiological sample (Troche, Wagner, Voelke, Roebers, & Rammsayer, 2014; Zhang et al., 2016). Gilbert, Akeroyd, and Gatehouse (2008) postulated that large variation in release time discrimination among participants with hearing impairment may have been influenced by cognitive ability.

## Methods

### Participants

**Figure 1.**
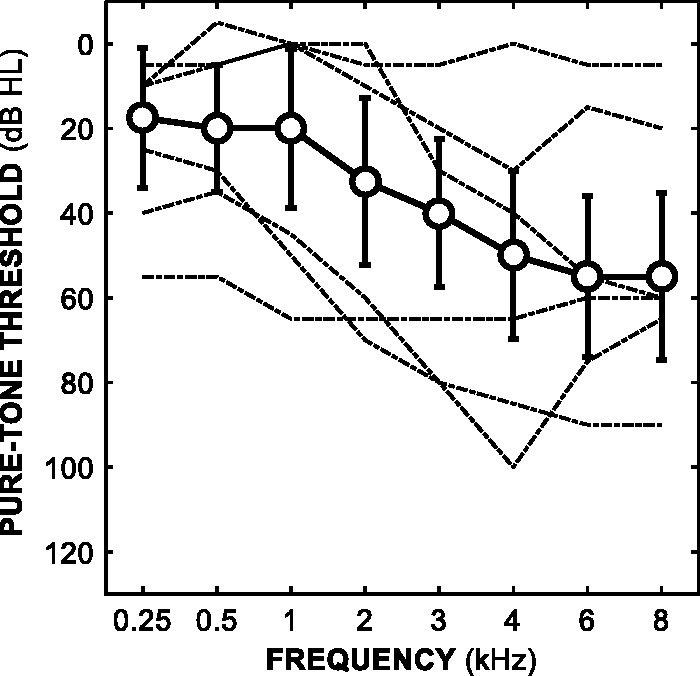
Median pure-tone thresholds as a function of frequency across all 38 participants. Error bars show ± 1 standard deviation. The dashed lines show the better-ear thresholds of participants with the three lowest and three highest BE4FA thresholds. BE4FA = better-ear four-frequency pure-tone average.

In total, 38 participants (14 females) were recruited from local audiology clinics. The median age of participants was 63.5 years, ranging from 37 to 74 years. Participants had varying degrees of hearing loss. Unmasked pure-tone thresholds were measured immediately prior to the experiment (British Society of Audiology, 2011). The median better-ear four-frequency pure-tone average (BE4FA, calculated as the average of thresholds at 0.5, 1, 2, and 4 kHz) was 30 dB HL, and ranged from 3 to 63 dB HL. Figure 1 shows the median audiogram across all 38 participants. Most participants had high-frequency sloping sensorineural hearing loss. Four participants had conductive elements to their hearing loss; this was based on differences between air and bone conduction thresholds exceeding 20 dB when averaged over three out of five frequencies at 0.5, 1, 2, 3, and 4 kHz (British Academy of Audiology, 2016). Eighteen participants were hearing-aid users; the median hearing-aid experience was 3 years, and ranged from 1 month to 36 years. In terms of proportion of life with a hearing-aid, the median experience was 0.04, and ranged from 0.004 to 0.80.

This study was approved by the West of Scotland research ethics service (WoS REC(4) 09/S0704/12). Informed written consent was obtained from all participants prior to the experiment.

### Stimuli

All stimuli were SSNs with gains independently digitally generated in MATLAB (version 9.0.0, The Mathworks, Inc., Massachusetts, USA) with a sampling rate of 44.1 kHz and sampling depth of 16 bits. Stimuli were presented monaurally via circumaural headphones (AKG K702, Vienna, Austria) to better (BE4FA) ears at 60 dB SPL prior to the application of gain.

**Figure 2.**
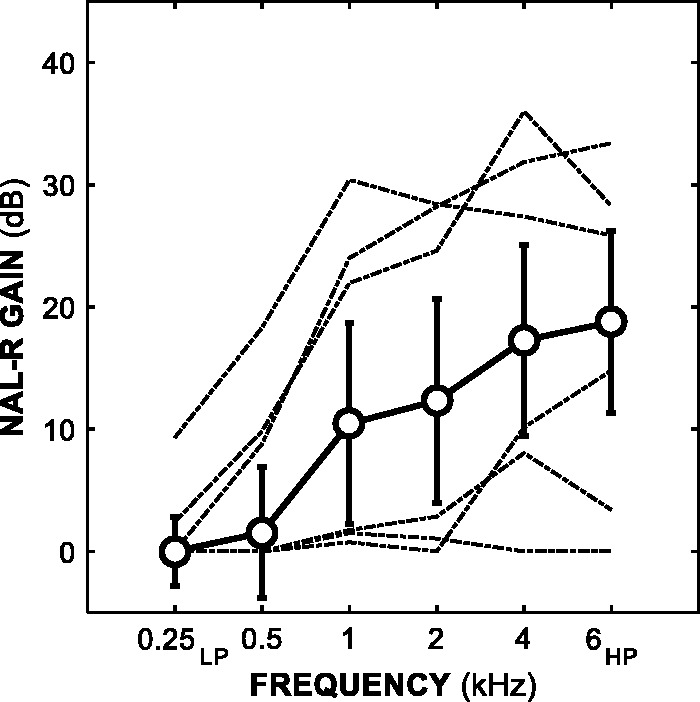
Median NAL-R prescription gains as a function of frequency across all 38 participants. Error bars show ± 1 standard deviation. LP refers to the 0.25 kHz low-pass band, while HP refers to the 6 kHz high-pass band. The dashed lines show the prescribed FGR curves of participants with the three lowest and three highest BE4FA thresholds. BE4FA = better-ear four-frequency pure-tone average; FGR = frequency-gain response.

Standard spectra were SSNs plus prescribed gains. The speech spectra were based on averaged male and female speech from 12 languages (Byrne, Dillon, & Tran, 1994). Gains were prescribed by applying the NAL-R formula (Byrne & Dillon, 1986; Dillon, 2012) to the audiogram for each participant’s better (BE4FA) ear. While NAL-R is an older formula, it provides a sufficient FGR baseline for linear gain adjustments, and also prescribes similar gains to more recent nonlinear formulae at the presentation level used in this study (Byrne, Dillon, Ching, Katsch, & Keidser, 2001; Dillon, 2012). Prescribed gains were applied to six frequency bands: a low-pass (LP) band with a cut-off frequency of 0.25 kHz, four octave-wide bands centered at 0.5, 1, 2, and 4 kHz, and a HP band with a cut-off frequency of 6 kHz. Figure 2 shows the median NAL-R prescription FGR across all 38 participants. Alternate spectra for the frequency-band conditions were SSNs plus prescribed gains, plus a fixed-level increment (Δ*L*) of 3, 6, 9, or 12 dB in one of six frequency bands, which were the same bands to which the gains were applied. Alternate spectra for the broadband condition were SSNs plus prescribed gains plus a Δ*L* of 1, 2, 3, or 4 dB applied across all six frequency bands. To generate stimuli, standard and alternate spectra were first multiplied with the complex spectra of independently generated Gaussian noise in the frequency domain, and then converted into the time domain using an inverse Fourier transform.

Standard stimuli were calibrated (using a Bruel & Kjaer Artificial Ear 4152 and Sound Level Meter 2260, Nærum, Denmark) so that the overall A-weighted presentation level was 60 dB SPL prior to the application of gain. Alternate stimuli were calibrated to confirm the Δ*L*s. Audibility was subjectively checked with participants after practice trials. The duration of each stimulus was 500 ms (including 20 ms raised cosine onset and offset ramps), separated by silent interstimulus intervals (ISIs) of 375 ms. Presentation level was roved across trials by a randomized amount chosen from a flat distribution of ± 3 dB.

There were four possible stimulus combinations for each trial: two same (standard–standard or alternate–alternate) and two different (standard–alternate or alternate–standard). Stimulus combinations were counterbalanced and presented randomly.

### Procedure

The experiment was conducted in a single, one-and-a-half–hour session. A screening questionnaire of basic patient details including hearing-aid status was completed, and unmasked pure-tone thresholds were measured. Cognitive ability was estimated after pure-tone audiometry. Following this, participants started the experimental task of discriminating frequency-band and broadband increments in SSNs. Participants were seated in a sound-proof audiometric booth, and stimuli were presented monaurally to their better-hearing ear. Participants completed two blocks of trials with a break between, each lasting approximately 15 to 20 minutes. Twenty practice trials were embedded into the start of each block.

A fixed-level, same-different task was utilized. Participants were asked to listen to each presentation and decide “Were the sounds the same or different?” Participants responded by choosing the appropriate button (“same” or “different”) on a touch screen monitor. Visual feedback (“correct” or “incorrect”) was provided following each response.

Twenty-eight Δ*L*s were presented: four Δ*L*s (3, 6, 9, and 12 dB) for each of the six (0.25 kHz_LP_, 0.5–2 kHz octave and 6 kHz_HP_) frequency-band conditions, and four Δ*L*s (1–4 dB) for the single broadband condition. Coupled with four stimulus combinations (two same, two different) repeated twice, there were 224 trials (excluding practice trials), presented in randomized order, per block. Each participant completed two blocks, resulting in 64 trials per psychometric function per participant for each of the seven conditions.

In a pilot study, we measured increment and decrement JNDs—negative adjustments to the prescription FGR—of 26 participants using a one-up, three-down adaptive, three-interval, 3AFC task. Decrement JNDs were difficult to measure; 21% of the total were excluded because of ceiling effects, poor adaptive tracks (the standard deviations of the final four reversals were 3 dB or greater), and failed adaptive tracks which terminated without a threshold estimate. While it is possible that this difficulty was influenced by the procedure, previous research has also reported difficulties in measuring decrement discrimination, which is suggested to be poorer than increment discrimination because of coding differences (Moore et al., 1989; Rinne, Sarkka, Degerman, Schroger, & Alho, 2006). Therefore, this current study only examines increment discrimination.

### Cognitive Tasks

Visual letter monitoring (VLM) and visual digit monitoring (VDM) tasks were used to estimate cognitive ability. Both tasks incorporate working memory, attention, reaction time, and continuous performance processes, and have been used previously in auditory research (Cox & Xu, 2010; Foo, Rudner, Rönnberg, & Lunner, 2007; Gatehouse et al., 2006; Knutson et al., 1991; Lunner & Sundewall-Thoren, 2007; Rudner, Foo, Sundewall-Thoren, Lunner, & Rönnberg, 2008).

In the VLM task, single consonants and vowels were presented alternately on a touch screen monitor, and participants identified three-letter consonant–vowel–consonant words. In the VDM task, single digits were presented, and participants identified even–odd–even sequences. Participants completed two runs for each task with ISIs of 1,000 and 2,000 ms, respectively. Participants completed a practice run with a 2,000-ms ISI prior to the formal runs. The task order was randomized across participants, although a 2,000-ms ISI run was always tested first. Correct hits and false alarms were measured and cognitive scores were expressed with *d’* values. A single *d’* score was aggregated across test types and speeds for each participant. Five participants—who were either dyslexic or nonnative English speakers—did not complete the tasks.

### Analysis

Discrimination in the same-different task was expressed with *d’* values for each Δ*L*. *d’* is a measure of sensitivity approximately linearly associated with signal strength, which increases with hit rate and decreases with false alarm rate (Klein, 2001; Macmillan & Creelman, 2005). *d’* can also be seen as procedure-free as it varies according to the number of stimulus presentations within a trial and percent correct values in forced choice tasks. Logistic functions were fit to *d’* values, and thresholds—JNDs—were estimated based on line fits to *d’* = 1. Thresholds associated with *d’* = 1 were estimated to provide a JND commensurate with other psychophysical research as well as a baseline for what is the minimally discriminable adjustment in our optimal listening conditions. For a fixed-level, same-different task where the participant is unbiased, *d’* = 1 is approximately equal to 55% correct (assuming a differencing strategy), corresponding to 76% correct in a 2AFC task (Green & Swets, 1966; Macmillan & Creelman, 2005).

These analyses were performed with equations and routines from Macmillan and Creelman (2005) and the Palamedes Toolbox (Prins & Kingdom, 2009). Our calculations assumed that participants adopted a differencing strategy of discrimination (Macmillan & Creelman, 2005). We estimated bias using false alarm rates, which were substituted into iterative calculations to estimate sensitivity. The log-linear rule correction factor for extreme data was applied across the dataset (Hautus, 1995; Stanislaw & Todorov, 1999), in which 0.5 was added to the number of hits and false alarms, and 1.0 was added to the number of targets.

**Figure 3.**
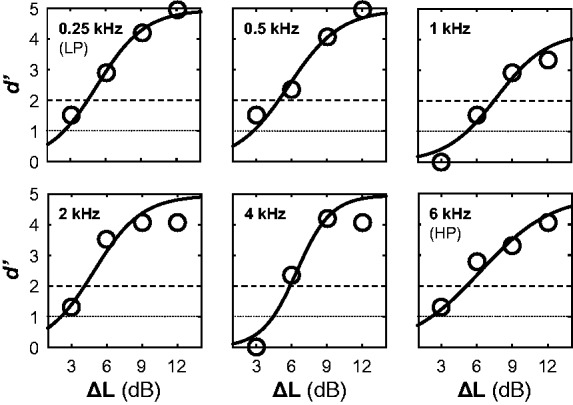
Example psychometric functions from a single participant’s six frequency-band conditions. The solid line shows logistic functions fit to *d’* data, represented as circles. The dotted line shows the *d’* = 1 threshold, while the dashed line shows the *d’* = 2 threshold.

Two types of JNDs were estimated: increment JNDs in six frequency bands (0.25 kHz_LP_, 0.5–2 kHz octave and 6 kHz_HP_) and broadband (across frequencies) increment JNDs. JNDs were determined by fitting logistic functions to *d’* data, with a JND corresponding to the Δ*L* dB estimated at line fits to *d’* = 1. Figure 3 shows examples of the psychometric functions calculated.

The Shapiro–Wilk test (Shapiro & Wilk, 1965) indicated that JNDs were not normally distributed. This was the case for frequency-band JNDs (*W* = 0.90; *p* < .001), as well for broadband JNDs (*W* = 0.90; *p* < .01). We therefore report median JNDs and nonparametric inferential statistical analyses for JND comparisons. Twenty-three JNDs (approximately 9% of the total) were excluded because of poor fits resulting in extreme values. These were identified by visualizing fits and assessing their associated root-mean-square error values.

### JNDs for Noisier Conditions

We additionally estimated JNDs as above at *d’* = 2, which corresponds to greater sensitivity for discrimination in noisier conditions, both acoustically and experimentally. We assume that JNDs at *d’* = 1 measured in noisy conditions would be greater (poorer) than the current JNDs at *d’* = 1. Considering this, we additionally report JNDs estimated at a more sensitive *d’* = 2, given the greater sensitivity required to discriminate in noisier conditions relative to the sensitivity required to discriminate in optimal conditions. This was done considering the increasing availability of technologies which allow for self-adjustments to highly complex stimuli in highly variable environments (Keidser & Convery, 2016; Nelson et al., 2018). For a fixed-level, same–different task where the participant is unbiased, *d’* = 2 is approximately equal to 68% correct, which corresponds to 92% correct in a 2AFC task (Macmillan & Creelman, 2005). Thirteen JNDs at *d’* = 2 (approximately 5% of the total) were excluded because of poor fits resulting in extreme values.

## Results

### Just-Noticeable Differences

**Figure 4.**
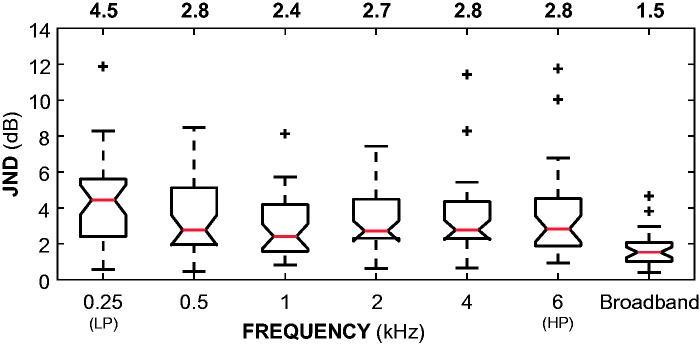
Box plots of JNDs (*d’* = 1) across all 38 participants. Notches on each interquartile-range display the 95% confidence interval around the median (red line). Median JNDs for each condition are displayed above the plots. Confidence limits that are greater than quartiles suggest uncertain medians. Whiskers extend to the most extreme JNDs that are within 1.5 × the interquartile range. Pluses indicate outliers outside 1.5 × the interquartile range. JND = just-noticeable difference.

Figure 4 shows JNDs at *d’* = 1. A Wilcoxon (1945) signed-rank test of paired samples with Holm–Bonferroni corrections for multiple comparisons (Holm, 1979) revealed no significant differences between frequency-band JNDs, except between the 0.25 kHz_LP_ and 1 kHz JNDs (*Z* = 2.80; *p* < .001). Because of this difference, the 0.25 kHz_LP_ JND is reported separately from the “frequency-band JND” grouping. Across all participants, all octave bands (0.5–4 kHz) and the 6 kHz_HP_ band, the median JND was 2.8 dB, 95% CI [2.5, 3.0]. For the 0.25 kHz_LP_ band, the median JND was 4.5 dB, 95% CI [3.7, 5.2]. The median broadband JND was 1.5 dB, 95% CI [1.2, 1.8]. Frequency-band JNDs were significantly greater than broadband JNDs (*Z* = 4.40 and 3.59 for the 0.25 kHz_LP_ and median frequency-band JNDs respectively;* p* < .001 for both).

Pearson correlation coefficients revealed a positive correlation between participants’ median frequency-band (across all except the 0.25 kHz_LP_ band) and broadband JNDs (*r* = 0.47; *p* < .01). Frequency-band JNDs were not correlated with each other when adjusting for multiple comparisons (all *p* > .05). There was only one significant correlation between frequency-band JNDs and pure-tone thresholds at the corresponding frequency when adjusting for correlations between thresholds and age: a positive correlation between participants’ 6-kHz threshold and their 6-kHz_HP_ JND (*r* = 0.39; *p* < .05). The 6 kHz_HP_ JNDs increased (i.e., were poorer) with increasing pure-tone threshold. There were no significant correlations between either BE4FA or age and individual JNDs (frequency-band or broadband) when adjusted for the correlation between BE4FA and age (all *p* > .05). A Wilcoxon rank-sum test revealed no significant differences between hearing-aid users’ and non–hearing-aid users’ median frequency-band and broadband JNDs (*p* > .05). There was no significant correlation between the proportion of life with a hearing-aid and median frequency-band or broadband JND (*p* > .05 for both).

### Cognitive Tasks

**Figure 5.**
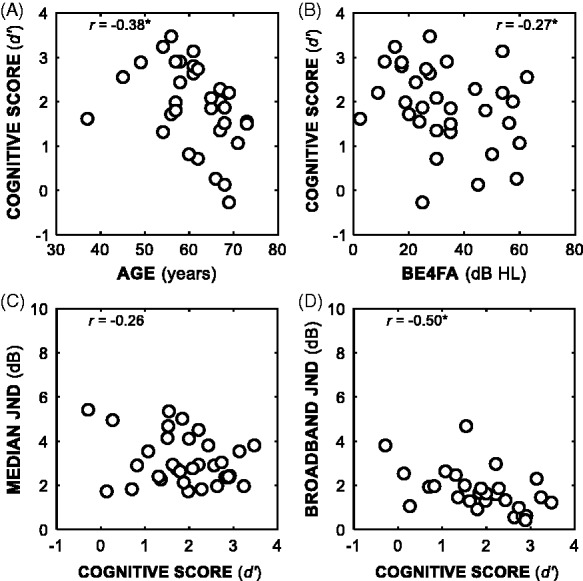
Scatterplots between individual cognitive scores and several variables: age (Panel A), BE4FA (Panel B), median frequency-band JND (calculated from all bands apart from the 0.25 kHz_LP_ band, Panel C), and broadband JND (Panel D). Correlations with asterisks were statistically significant (*p* < .05). BE4FA = better-ear four-frequency pure-tone average; JND = just-noticeable differences.

Across 33 participants (median age of 62 ± 8.0 standard deviation years), the average cognitive score was 1.9 out of a total of 3.6. Age was negatively correlated with cognitive score (*r* = −0.38; *p* < .05), as was BE4FA (*r* = 0.27; *p* < .05). We calculated standard coefficients as opposed to partial coefficients as neither age nor BE4FA correlated with JNDs. Frequency-band JNDs were not correlated with cognitive score (*r* = 0.26; *p* > .05). Broadband JNDs were negatively correlated with cognitive score (*r* = −0.50; *p* < .05). Figure 5 shows cognitive score scatterplots.

### JNDs for Noisier Conditions

**Figure 6.**
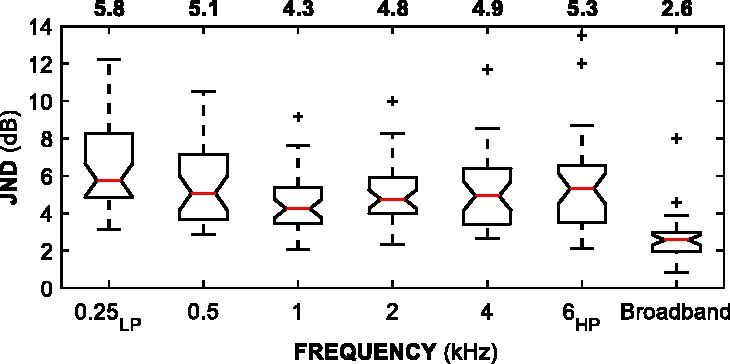
Box plots of JNDs (*d’* = 2) across all 38 participants. See Figure 4 for description.

Figure 6 shows additional JNDs estimated at *d’* = 2. Similar to the JNDs at *d’* = 1, the 0.25 kHzLP JNDs were significantly different to the 1-, 2-, and 4-kHz JNDs were (*Z* = 3.80, 2.62, and 2.76, respectively; all *p*<.05). In this dataset, the 0.5-kHz and 1-kHz JNDs were also significantly different (*Z* = 3.80; *p* < .01). The remaining JNDs comparisons were not significantly different. Across all participants, the median JND for higher frequency bands (1–4 kHz octave and 6 kHz_HP_) was 4.8 dB, 95% CI [4.4, 5.1]. The median JND for lower frequency bands (0.25 kHz_LP_ and 0.5 kHz octave) was 5.4 dB, 95% CI [4.6, 6.2]. The median broadband JND was 2.6 dB, 95% CI [2.2, 3.0]. Frequency-band JNDs were significantly greater than broadband JNDs (*Z* = 5.01 and 4.38 for lower and higher frequency bands, respectively;* p* < .001 for both).

## Discussion

We measured increment discrimination in six individual frequency-bands of SSNs. Participants had varying degrees of hearing impairment, and stimuli were presented at a baseline of 60 dB SPL plus individually prescribed gains. The frequency-band JND was 2.8 dB, and 4.5 dB for the 0.25 kHz_LP_ band. We identified a positive correlation between participants’ median frequency-band and broadband JNDs, suggesting some consistency between the discrimination of frequency-band and broadband increments.

These results are congruent with previous literature. Using a 2AFC task with a one-up, two-down adaptive procedure (converging on 71% correct, approximately *d’* = 0.77), Moore et al. (1989) measured a 1-kHz JND of 2.1 dB for three trained participants with normal hearing. In the current study, the 1-kHz JND was 2.4 dB. The similarity between these trained normal-hearing and our participants’ JNDs may be because of the NAL-R gains applied to our presentations; using a 4AFC task with a one-up, two-down adaptive procedure (converging on 71% correct, approximately *d’* = 1.52), Turner and Holte (1987) reported a 5-dB second-formant peak JND for participants with normal-hearing and also participants with hearing impairment who were prescribed with constant HP gain. This second-formant peak JND is similar to our 2-kHz JND of 4.8 dB at *d’* = 2.

JNDs did not correlate with BE4FA hearing ability. Correlations between frequency-band JNDs and pure-tone thresholds were absent apart from a positive correlation between 6 kHz pure-tone thresholds and 6 kHz_HP_ JNDs. It is possible that the gains prescribed at the 6 kHz_HP_ band may not have been sufficient for participants with more steeply sloping losses around 6 kHz, particularly when considering that the NAL-R formula includes an average of pure-tone thresholds at 0.5, 1, and 2 kHz as a factor. Participants with the most severe 6 kHz pure-tone thresholds had 6 kHz_HP_ JNDs greater than 10 dB, and removing a single of these elevated JNDs eliminates any statistically significant correlation.

The median broadband JND was 1.5 dB. Presenting stationary SSNs in a 2AFC one-up, three-down adaptive procedure (converging on 79% correct, approximately *d’* = 1.16), Whitmer and Akeroyd (2011) measured similar broadband JNDs of 1.3 dB and 1.0 dB with unaided and aided participants, respectively. These broadband JNDs were independent of BE4FA hearing ability and age, as with the current study. Dirks et al. (1993) and Jenstad et al. (2007) reported that participants could distinguish between FGR curves with an adjustment of 4.5 dB. It seems reasonable that this is greater than our broadband JND; the current study’s participants made discrimination judgments on SSNs in quiet, rather than preference judgments on speech in noise.

Lunner and Sundewall-Thoren (2007) reported an average VLM score of 2.0 with 23 hearing-aid users (mean age of 65.6 years). We measured an average VLM score of 1.8, suggesting that our samples were similar on this specific cognitive measure. Cognitive score was negatively correlated with age in the current study. Gatehouse et al. (2006) did not report a significant correlation using the same tasks, and neither did Foo et al. (2007) using a VLM task: When analyzing only VLM scores, our correlation becomes insignificant, suggesting that the VLM task may be a poorer predictor of age than the VDM task. Correlations between participants’ cognitive score and median frequency-band JNDs were insignificant. There was, however, a negative correlation between participants’ cognitive scores and broadband JNDs, suggesting that patients with poorer cognitive abilities may require greater broadband FGR adjustments to be noticeable. The frequency-band discrimination task may not have elicited similar cognitive processes to those elicited in the VLM and VDM tasks, particularly when considering that these cognitive tasks are visually based as opposed to aurally based. These tasks have not been used in examination with more basic auditory discrimination as in the current study. Previous research that investigated the link between auditory discrimination and cognitive ability utilized different tasks (Troche et al., 2014; Zhang et al., 2016), as did several studies which reported relationships between hearing-aid processing schemes and cognitive ability (Foo et al., 2007; Lunner, 2003; Rudner et al., 2008).

JNDs at *d’* = 1 suggest initial frequency-band adjustments of 3 dB to be immediately noticeable in a quiet clinic. The additional JNDs at *d’* = 2 suggest initial frequency-band adjustments of 5 dB to be immediately noticeable in noisier conditions. A hearing-aid user should not notice a difference if the FGR is adjusted by a value lower than a JND at the respective band. However, fine-tuning in the clinic as well as self-adjustments in less controlled environments are typically tested with speech, and therefore it would be appropriate to measure JNDs with speech stimuli in a future study. We expect speech JNDs to be greater than SSN JNDs given the highly complex spectro-temporal properties of speech. It is of note that the JNDs at *d’* = 2 mostly corroborate recently revised REM tolerances of ± 5 dB across frequencies (British Society of Audiology, 2018).

Stimuli in the current study were processed linearly, while most modern hearing aids utilize wide-dynamic range compression. Compression, which reduces gain with increasing input, should make increment discrimination more difficult. However, compression is usually applied prior to, or with the application of gain, and therefore should have a limited effect on discrimination of gain adjustments in the clinic. Furthermore, studies using clinically relevant compression ratios (between 1.1:1 and 2:1) have reported little difference between level-based discrimination measured with and without compression (Akeroyd, 2010; Whitmer & Akeroyd, 2011). In addition, evidence suggests that listeners with hearing impairment are only sensitive to compression adjustments under optimal listening conditions (Gilbert et al., 2008; Nabelek, 1984; Sabin, Gallun, & Souza, 2013). Musa-Shufani, Walger, Von Wedel, and Meister (2006) did report that interaural level difference JNDs measured with narrowband noises were greater when measured with compression than when measured linearly, although the compression ratios were strong (3:1 and 8:1). While measured with localization tasks, this study suggests that strong compression can affect level-based JNDs.

There was a fair amount of variance across participants, similar to previous research examining the detection and discrimination abilities of listeners with hearing-impairment (Gilbert et al., 2008; Lentz & Leek, 2003; MacPherson & Akeroyd, 2014; Turner & Holte, 1987). The variance in frequency-band JNDs was not predicted by age, BE4FA, or cognitive score, and was only predicted by pure-tone thresholds to a small extent. The variance in broadband JNDs was not predicted by age or BE4FA, although broadband JNDs did correlate with cognitive score, suggesting that some variance in level discrimination may be because of variances in cognitive ability. Unexplained variance among JNDs may have been influenced by unquantified cochlear or high-level processing deficits in participants.

## Summary

We measured frequency-band and broadband JNDs for gain increments in SSNs. Participants had varying degrees of hearing loss and were provided with prescribed amplification. Frequency-band JNDs at *d’* = 1 were mostly independent of centre frequency; the median frequency-band JND was 2.8 dB, and the 0.25 kHz_LP_ JND was 4.5 dB. The broadband JND was 1.5 dB. JNDs were also mostly independent of pure-tone threshold. Although frequency-band JNDs were greater than broadband JNDs, correlations suggest some relationship between frequency-band and broadband discrimination. While frequency-band JNDs were not correlated with cognitive score, broadband JNDs were. JNDs at *d’* = 1 suggest initial frequency-band gain adjustments of 3 dB to be noticeable in a quiet clinic. JNDs at *d’* = 2 suggest initial frequency-band gain adjustments of 6 dB for frequencies below 1 kHz, and 5 dB for frequencies at and above 1 kHz in noisier environments.
